# The Carboxy Terminal Region of the Human Cytomegalovirus Immediate Early 1 (IE1) Protein Disrupts Type II Inteferon Signaling

**DOI:** 10.3390/v6041502

**Published:** 2014-04-02

**Authors:** Bindu Raghavan, Charles H. Cook, Joanne Trgovcich

**Affiliations:** Department of Surgery, The Ohio State University, 473 West 12th Avenue, Columbus, OH 43210, USA; E-Mail: bindu.raghavan@monsanto.com (B.R.); Charles.Cook@osumc.edu (C.H.C.)

**Keywords:** human cytomegalovirus, interferons, interferon γ, immediate early 1, IE1, STAT1, STAT2, ND10, antiviral defense

## Abstract

Interferons (IFNs) activate the first lines of defense against viruses, and promote innate and adaptive immune responses to viruses. We report that the immediate early 1 (IE1) protein of human cytomegalovirus (HCMV) disrupts signaling by IFNγ. The carboxyl-terminal region of IE1 is required for this function. We found no defect in the initial events in IFNγ signaling or in nuclear accumulation of signal transducer and activator of transcription 1 (STAT1) in IE1-expressing cells. Moreover, we did not observe an association between disruption of IFNγ signaling and nuclear domain 10 (ND10) disruption. However, there is reduced binding of STAT1 homodimers to target gamma activated sequence (GAS) elements in the presence of IE1. Co-immunoprecipitation studies failed to support a direct interaction between IE1 and STAT1, although these studies revealed that the C-terminal region of IE1 was required for interaction with STAT2. Together, these results indicate that IE1 disrupts IFNγ signaling by interfering with signaling events in the nucleus through a novel mechanism.

## 1. Introduction

After primary infection, human cytomegalovirus (HCMV) persists for the lifetime of the host, avoiding elimination by the host immune system [1]. In congenitally infected infants and immunosuppressed persons, HCMV can cause serious diseases [2]. Aspects of both HCMV disease and persistence in human populations are suspected to be linked to the multiple mechanisms this virus has evolved to modulate human immune responses. 

Interferons direct intrinsic cellular defenses and early immune responses against HCMV and other viruses. IFNs are classified into three categories: type I (IFNs-α, β, ω, ε, κ), type II (IFNγ) and type III (IFNl), though type I and II interferons are the best studied with respect to their antiviral functions [3]. IFNs have intrigued virologists for over 50 years, not only for providing insight into viral biology and the molecular combat that occurs in infected cells, but also for the potential promise of exploiting the IFN system for therapeutic control and prevention of viral diseases. IFNs function to limit virus replication and pathogenesis through stimulation of intrinsic cellular defense mechanisms that contribute to innate immunity [4,5], and by promoting and regulating adaptive immune responses [6,7]. 

The antiviral functions of IFNs are initiated by binding with their receptors [4,8,9,10]. The type I receptor is composed of two proteins, IFN Alpha Receptor 1 (IFNAR1) and IFN Alpha Receptor 2 (IFNAR2). The cytoplasmic tails of the receptors associate with protein tyrosine kinases, Janus Kinase 1 (JAK1) and Tyrosine Kinase 2 (TYK2). Binding of Type I IFN to its cognate receptor leads to phosphorylation of JAK1 and TYK2 and tyrosine residues on the cytoplasmic tails of the receptors. Phosphorylation of the receptors enables docking of Signal Transducer and Activator of Transcription 1 and 2 (STAT1 and STAT2) via Src Homology 2 (SH2) domains and tyrosine phosphorylation and heterodimerization of the STATs. The STAT1-STAT2 heterodimers forms complexes with interferon regulatory factor (IRF)9 that translocates to the nucleus and binds Interferon Stimulated Response Elements (ISRE) in the promoters of type I IFN induced genes [8,9]. Type II IFNs bind to a different receptor composed of Interferon Gamma Receptor 1 and 2 (IFNγR1 and IFNγR2) proteins, associated with the tyrosine kinases JAK1 and JAK2. IFNγ signaling results in formation of phosphorylated STAT1 homodimers that translocate to the nucleus and bind Gamma Activated Sequence (GAS) elements in the promoters of type II IFN inducible genes [3,9,10].

Interferons can stimulate the expression of over 300 cellular genes known as Interferon Stimulated Genes (ISGs) [11]. The proteins encoded by ISGs have antiviral and immunomodulatory activity. These gene products interfere with the viral life cycle by degrading viral nucleic acids, inhibiting viral protein synthesis, interference with viral protein trafficking, or inducing apoptosis leading to death of virally infected cells [10]. In addition to their direct antiviral role, IFNs can control viral pathogenesis by enhancing adaptive immune responses, enhancing antigen presentation to cells of the immune system, and recruiting immune cells to sites of infection [6,7]. In particular, type II IFN is important in stimulating the activity of NK cells, macrophages, T lymphocytes and dendritic cells (DCs) [7]. 

The cytomegaloviruses employ multiple mechanisms to disrupt IFN induction [12,13,14] and IFN signaling [15,16,17,18,19,20]. HCMV targets the type I pathway by disrupting STAT1 and STAT2. Paulus and colleagues demonstrated that HCMV immediate early 1 (IE1/IE72) encoded by the *UL123* gene forms a physical complex with STAT1 and STAT2 thereby blocking signaling after nuclear translocation and before DNA binding [21]. Binding of IE1 to STAT2 requires the short acidic and serine/proline-rich low-complexity motifs in the carboxy-terminal region of IE1 [22]. Huh and colleagues demonstrated that disruption of IFNβ activity related to binding the acidic domain of immediate early 1 (IE1) to STAT2 in a sumoylation-dependent manner [23]. Le *et al.* further reported that STAT2 is targeted for proteasome-mediated degradation at early to late times of infection that was dependent on expression of an early gene [24]. 

The type II IFN signaling pathway has been studied in both MCMV- and HCMV-infected cells. A blockade in IFNγ-mediated regulation of several genes is observed in murine cytomegalovirus (MCMV) infected macrophages [25], and the MCMV M27 protein has been shown to disrupt IFNγ signaling through a novel, STAT2-dependent mechanism [26]. Initial reports that HCMV also targets type II IFN signaling derived from the observation that IFNγ-induced CIITA induction was disrupted downstream of STAT1 nuclear translocation as early as 6 hours after HCMV infection [27]. This appeared to be due to impaired binding of STAT1 to GAS elements at very early times in HCMV infected cells [28]. Subsequently, it was reported that IFNγ signaling in HCMV infected cells is also disrupted through degradation of JAK1 [29]. Furthermore, Baron and Davignon described impaired STAT1 tyrosine phosphorylation in response to IFNγ in the 12 to 24 hour time period after infection with HCMV [30]. This was found to be linked to activation of the SH2 domain-containing phosphatase 2 (SHP2) acting on phosphorylated STAT1. Remarkably, Knoblach and colleagues described an activation of a type II interferon-like host response in cells induced to express IE1 [31]. The activation ISGs by IE1 in this report was attributed to activation of STAT1 and was independent of IFNγ. Altogether there is a lack of clarity on how HCMV influences type II IFN signaling and the viral gene products involved.

Here we report that expression of the HCMV *UL123* gene that codes for IE1/IE72 also interferes with IFNγ signaling in human primary fibroblasts. IE1 is a promiscuous transactivator of viral and cellular genes [32] and, as discussed above, is known to antagonize type I interferon signaling [21,23,24]. Our findings suggest that IE1 can disrupt signaling by both type I and Type II interferons. Furthermore we have determined that the carboxyl-terminal region of IE1 that includes the acidic domain is required for this function. We found no defect in the initial events in IFNγ signaling in IE1‑overexpressing cells, nor did we observe an association between disruption of IFNγ signaling and ND10 disruption. Moreover, IE1 does not interfere with nuclear accumulation of STAT1. However there is reduced binding of STAT1 homodimers to target GAS elements in the presence of IE1. This activity does not appear to require a direct interaction of IE1 and STAT1 and suggests that IE1 disrupts IFNγ signaling in the nucleus and through a novel mechanism. 

## 2. Results and Discussion

### 2.1. The HCMV IE1 Gene Disrupts Signaling by Type II Interferon

To identify the HCMV genes involved in disruption of IFN signaling a cDNA library of the HCMV laboratory strain AD169 was constructed [33]. The human fibrosarcoma cell line 2C4 was used in a preliminary screen for HCMV cDNA clones that have a role in regulating IFN signaling. 2C4 is a fibrosarcoma cell line engineered to express the T-cell antigen CD2 under the control of the promoter element of the Interferon Induced Transmembrane protein 1 (*IFITM1*) gene (a.k.a. 9-27, IFI17, CD225) which respond to both type I and II IFNs by increasing cell surface expression of CD2 [34]. 2C4 cells transfected with any HCMV cDNA clone involved in disruption of IFN signaling would thus be expected to exhibit reduced accumulation of cell surface of CD2. Using this system we observed that transfection of cDNA clone pIE622, harboring the full length cDNA sequence for the HCMV *UL123* gene, was associated with diminished CD2 cell surface levels relative to empty vector-transfected cells after exposure to IFNβ, and to a lesser extent, IFNγ (data not shown). This suggested a role for IE1 in disruption of both type I and type II IFN signaling. The *UL123* gene codes for the HCMV major transcriptional transactivator protein, IE1. In addition to its role as a promiscuous transactivator of viral and cellular genes, IE1 is known to have multiple functions including disruption of ND10 nuclear bodies [35], antagonism of histone deaceytlase (HDAC3) [36], anti-apoptotic function [37], chromatin tethering [38], and interference with signaling by type I interferons [21]. 

**Figure 1:**
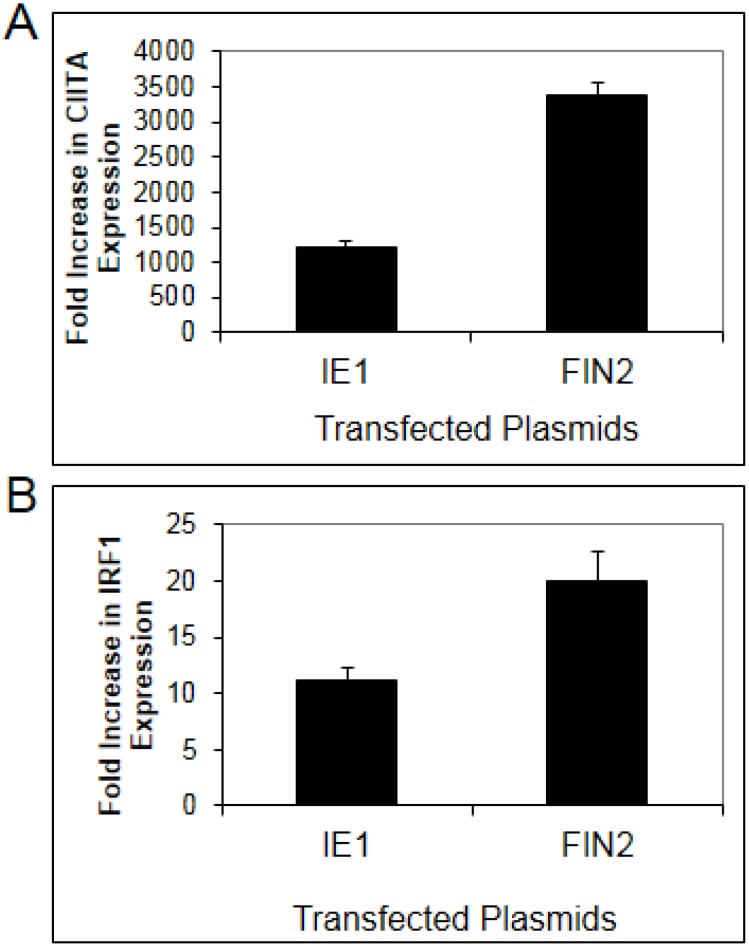
Diminished interferon-induced gene expression in human fibroblasts expressing IE1. MRC-5 fibroblasts were nucleofected with plasmid pIE622 harboring the *UL123* gene specifying IE1, or the pFIN2 empty vector. At 24 hours post-nucleofection, the cells were exposed to 100 U/mL of IFNγ or were left untreated. At 6 hours after treatment, total RNA was isolated and subjected to Real Time RT-PCR analysis as described in Materials and Methods. The fold increase in CIITA (**A**) (*p* value = 0.053) or IRF1 (**B**) (*p* value 0.017) transcript levels in IFN treated cells relative to untreated cells was determined by the ΔΔCt method. Data shown are the average of three independent experiments.

To confirm a role for IE1 in disruption of type II IFN signaling, we examined the effect of IE1 in a more physiologically relevant human fibroblast cell line. Basal expression of MHC II Transcriptional Activator (CIITA) is very low in fibroblasts but becomes highly induced after IFNγ treatment. CIITA transcript levels were quantified by Real-Time RT-PCR in MRC5 fibroblasts after nucleofection with pIE622 expressing the full length IE1. In MRC5 cells, nucleofection resulted in greater than 90% transfection efficiency (data not shown). CIITA mRNA levels were compared in IFNγ treated cells and untreated cells. In untreated cells, expression of IE1 was associated with a slight induction of CIITA or IRF1 (0.9 to 3 fold). In IFNγ treated cells, we found that CIITA induction in IE1 expressing cells was only 39% of that observed in cells nucleofected with empty vector (Figure 1A). We also examined the IFNγ-induced expression of a second gene, IRF1, in IE1 expressing fibroblasts. We found that the increase in expression of IRF1 in response to IFNγ is reduced by 52% in IE1 expressing cells as compared to cells nucleofected with empty vector (Figure 1B) relative to untreated cells. These findings confirm that IE1 is able to impair, but not completely block IFNγ-induced upregulation of CIITA and IRF1 transcript levels in human fibroblasts.

### 2.2. Mapping of the IE1 Protein Region Involved in Disruption of IFN-Induced Gene Expression

**Figure 2:**
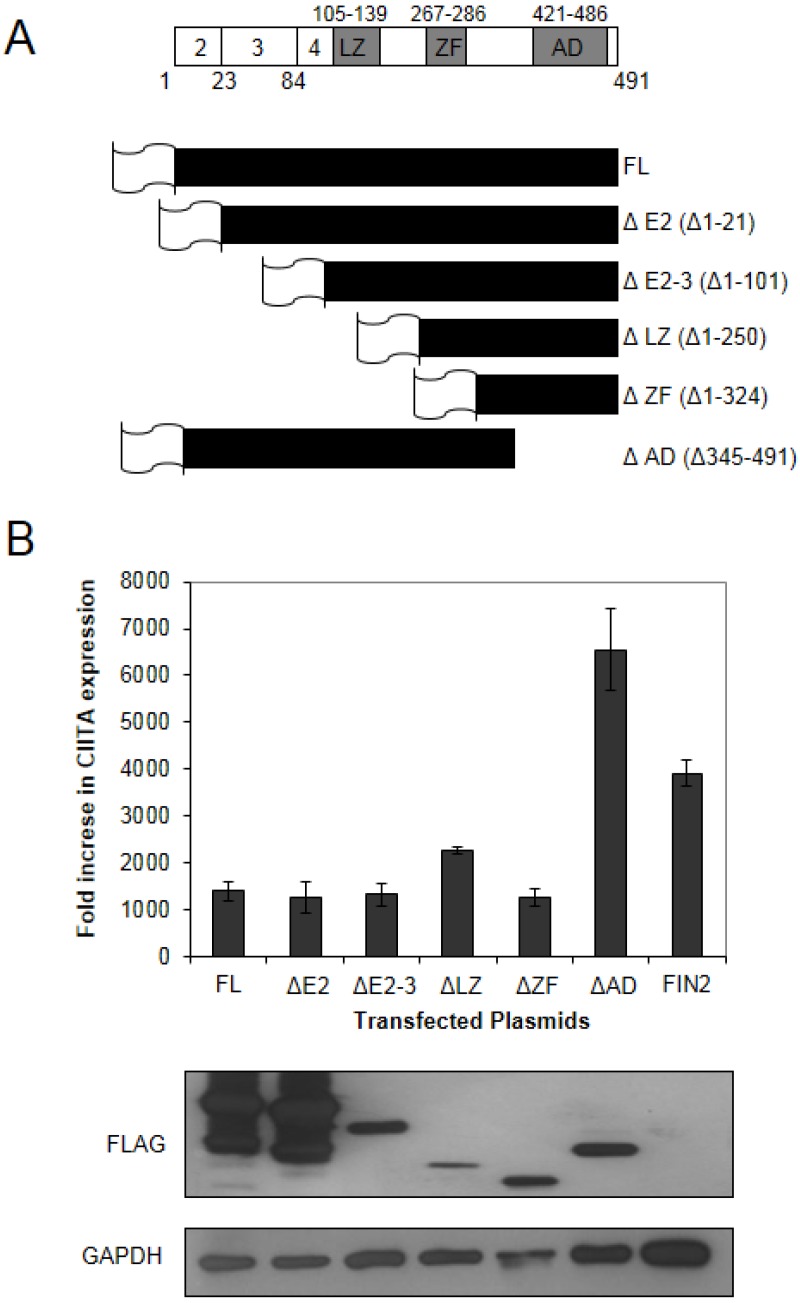
The carboxy-terminal region of IE1 is required for disruption of IFNγ signaling. (**A**) Schematic of the IE1 protein and truncation mutants generated in this study. Top line: a schematic of the IE1 protein indicating the amino acid positions comprising the coding exons (below) and the positions of the known functional domains (above). The coding exons are shown in white boxes. The functional domains of exon 4 are shown in shaded boxes and include the leucine zipper (L), the zinc finger (ZF) and the acidic domain (AD). A schematic of the five different IE1 N-terminal truncation mutants generated in this study are shown below. The sixth mutant is a C-terminal truncation mutant. The designation of the plasmids harboring the truncation mutants is based on the deletion of relevant exons or functional domains shown on the right, and the deleted amino acid residues are shown in parentheses. The gene sequences were inserted in frame to sequences specifying a FLAG epitope (depicted as a flag). (**B**) Interferon Stimulated Gene (ISG) induction in fibroblasts expressing truncated IE1 proteins**. **The indicated plasmids were nucleoporated into MRC5 cells and 24 hours after nucleofection the cells were treated with 100 U/mL IFNγ. At 6 hours after treatment total RNA was isolated. Real Time RT-PCR analysis was carried out for CIITA and 18S rRNA (endogenous control). The fold increase in CIITA transcript levels in IFN treated cells relative to untreated cells was determined by the ΔΔCt method. Shown is the average of two independent experiments. A film image of immunoblot analysis is shown below. In replicate cultures of the first experiment, cells were treated as above and solubilized. Equal amounts of protein from each lysate were subjected to electrophoresis in a denaturing polyacrylamide gel. Proteins were transferred to nitrocellulose sheets and reacted with the indicated antibodies. Anti-FLAG antibody was used to detect the presence of FLAG tagged IE1 proteins and antibody to GAPDH was used to evaluate protein loading.

The HCMV IE1 protein is a multifunctional protein with various functions attributed to different domains of the protein (Figure 2A). We generated a series of FLAG epitope-tagged truncation mutants to identify the protein region required for attenuation of IFNγ-induced gene expression as described in Materials and Methods. We generated five different FLAG-tagged truncation mutants plus a full length FLAG-tagged version of IE1 (Figure 2A). These six different clones were nucleofected into MRC5 cells, followed by treatment with IFNγ for 6 hours. RNA extracted from these cells was analyzed for levels of CIITA transcript induction by Real-Time RT-PCR. We found that there was a reduction in the IFNγ-induced expression of CIITA in cells expressing full length and truncated versions of IE1, with the exception of the C-terminal truncation missing residues 345–491 (ΔAD) that includes the acidic domain and chromatin tethering domain (Figure 2B). We consistently observed higher levels of CIITA induction in cells expressing the Δ345–491 protein relative to our vector alone transfections. Although the reason for this is not clear, it may be that the IE1 protein without the C-terminal domain provides a signal that amplifies IFNγ-induced CIITA gene expression. This may also relate to the report linking inducible IE1 expression to STAT1-dependent interferon-like gene induction [31]. One possibility is that IE1 may interact with cellular proteins that stimulate a type II interferon response, but this activity is masked by the dominant inhibitory role of the C-terminal 147 residues in our studies. These contradictory data may also reflect the different transfection systems or viral strains used in these two studies.

### 2.3. Disruption of IFNγ Signaling by IE1 Does Not Involve Interaction with PML

**Figure 3:**
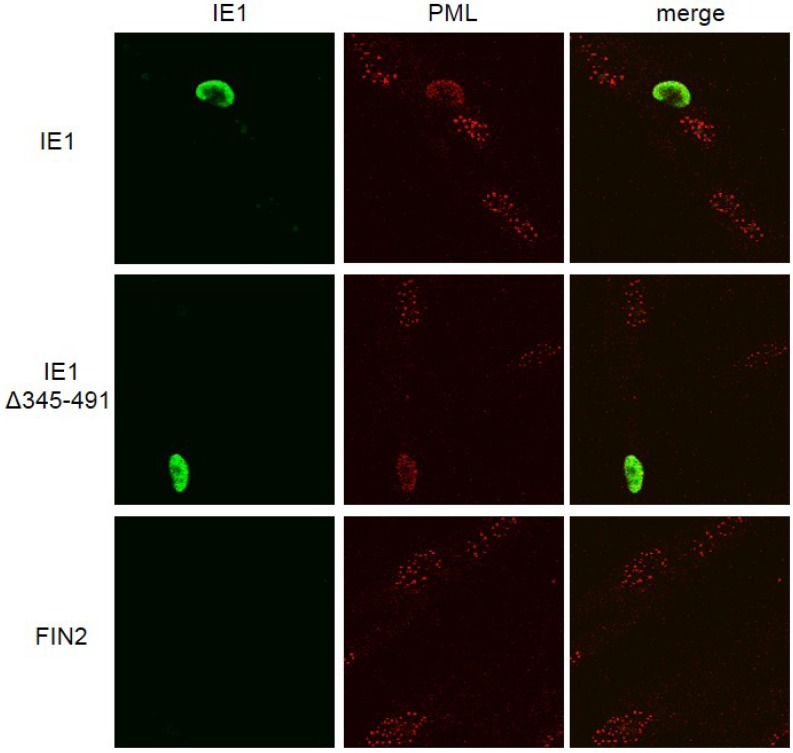
Disruption of ND10 structures in cells expressing IE1 and the C-terminal truncation mutant. Immunofluorescence images of IE1 and Promyelocytic leukemia protein (PML) in fibroblast cells. MRC5 cells were nucleofected with the indicated constructs. At 24 hours after nucleofection cells were fixed in methanol and reacted with mouse monoclonal anti-IE1 antibody (Mab810) followed by anti-mouse alexafluor 488 as secondary antibody. The cells were then exposed to rhodamine conjugated anti PML antibody (PGM3). IE1 reactivity is visualized in green (left column), PML reactivity is visualized in red (middle column) and merged images are shown in the right column.

Promyelocytic leukemia protein (PML) is involved in transcriptional repression and is a master organizer of nuclear domain (ND)10 structures. Since IE1 has been reported to be involved in the disruption of ND10 structures in the nucleus [35,39,40,41,42,43] and because PML is known to mediate antiviral activities of IFNs in HSV-infected cells [44] and to confer intrinsic immunity against CMV [45,46], we set out to test the hypothesis that the function of IE1 in disrupting IFNγ-induced gene expression is linked to its role in disrupting ND10 structures by immunofluorescence microscopy. We found that in fibroblasts expressing the full length IE1 and the Δ345–491 C-terminal deletion mutant of IE1, PML was dispersed throughout the nucleus, indicating disruption of ND10s (Figure 3). As expected, PML staining was in the form of punctate dots in the nucleus in the absence of IE1. Therefore, the C-terminal deletion mutant retains the ability to disrupt ND10s. We conclude that IE1‑mediated disruption of the IFNγ signaling pathway is not strictly linked to IE1-mediated dispersal of ND10s.

Earlier there have been conflicting reports, one suggesting that the acidic domain is important for PML targeting and disruption of ND10 structures [41] and the other suggesting that the acidic domain is not involved in this function of IE1 [39]. Yet another study revealed an intermediate role for the acidic domain IE1 wherein it was required to target ND10 structures but could not disrupt them [47]. Our results indicate that a C-terminal truncation mutant that includes the entire acidic domain is able to disrupt ND10s similar to the full length IE1 protein in MRC5 cells. Our results are in agreement the findings of Wilkinson *et al.* [39]. Although these results to do not preclude a role for PML in IE1‑mediated IFN signal disruption, they do demonstrate that this function of IE1 does not involve disruption of ND10 structures.

### 2.4. IE1 Does Not Perturb Initial Events in IFNγ-Mediated Signal Transduction

**Figure 4:**
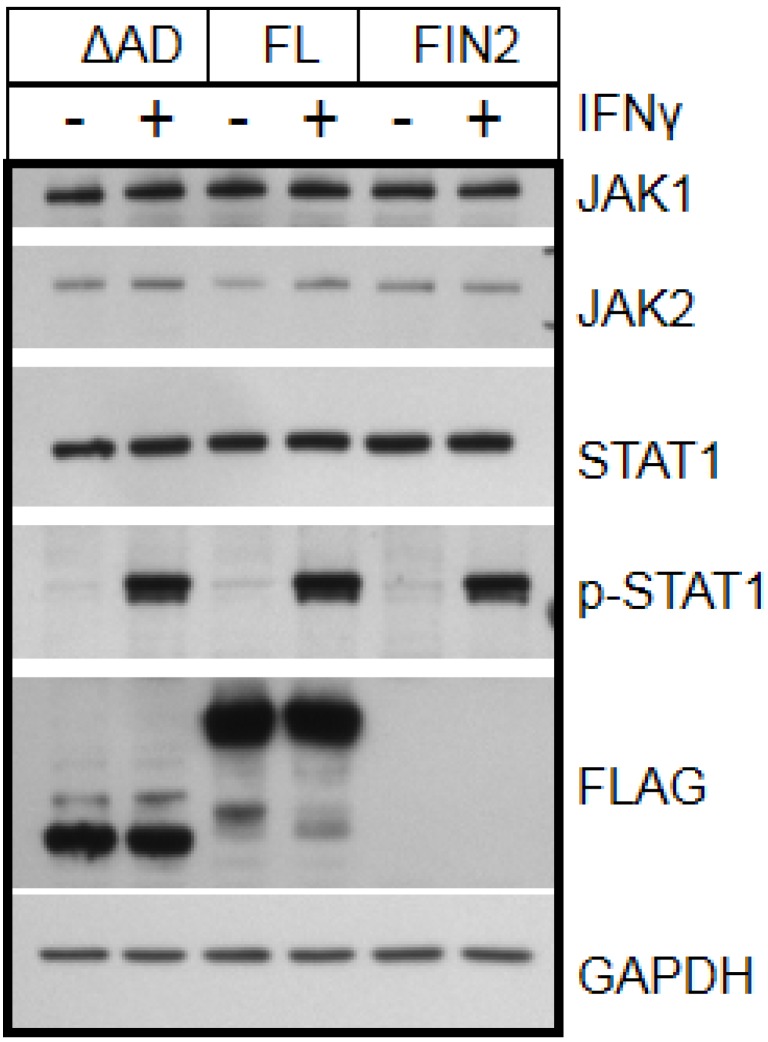
IE1 does not alter the state or abundance of key signaling molecules of the IFNγ pathway. Film images of electrophoretically separated cell lysates reacted with antibodies to IFNγ pathway signaling components. MRC5 cells were nucleofected with plasmid harboring full length IE1 (FL), the Δ345–491 C-terminal truncation IE1 (ΔAD) or empty vector (FIN2). At 24 hours after nucleofection, cells were exposed to IFNγ for 30 minutes or left untreated. Cells were solublized and equal amounts of protein from each lysate were subjected to electrophoresis in a denaturing polyacrylamide gel. Proteins were transferred to nitrocellulose sheets and reacted with the indicated antibodies. Anti-FLAG antibody was used to detect the presence of FLAG tagged IE1 proteins and antibody to GAPDH was used to evaluate protein loading.

**Figure 5:**
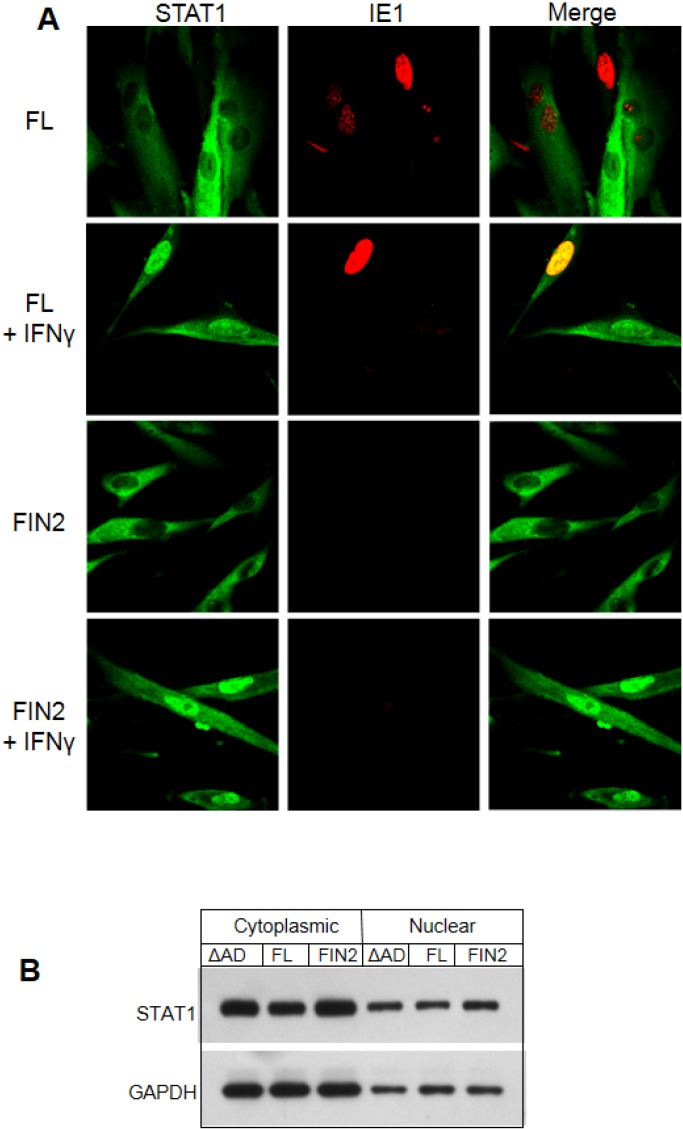
Nuclear translocation of STAT1 is not altered by IE1. (**A**) Immunofluorescence images of IE1 and STAT1 in cells with and without IFNγ treatment. MRC5 cells were nucleofected with the full length IE1 (FL) or empty vector (FIN2). After 24 hours cells were exposed to IFNγ for 30 minutes or left untreated. Cells were fixed in methanol and reacted with mouse monoclonal anti-IE1 antibody (Mab810) and rabbit polyclonal anti‑STAT1 antibody followed by anti-mouse alexafluor 543 and anti-rabbit alexafluor 488 as secondary antibodies. STAT1 reactivity is visualized in green (left column), IE1 reactivity is visualized in red (middle column) and merged images are shown in the right column. (**B**) Film images of electrophoretically separated cell lysates reacted with antibodies to STAT1 and GAPDH (loading control). MRC5 cells were nucleofected with plasmid harboring full length IE1 (FL), the Δ345–491 C-terminal truncation IE1 (ΔAD) or empty vector (FIN2). At 72 hours after nucleofection, cells were exposed to IFNγ for 30 minutes or left untreated. Cells were harvested and nuclear and cytoplasmic fractions were separated. Equal amounts of protein from the nuclear and cytoplasmic lysates were subjected to electrophoresis in a denaturing polyacrylamide gel. Proteins were transferred to nitrocellulose sheets and reacted with the indicated antibodies.

We next examined the state and abundance of proteins involved in IFNγ signaling in IE1-expressing cells. We found no difference in the levels of total JAK1, JAK2 and STAT1 in IE1-expressing cells as compared to cells expressing the Δ345–491 mutant or cells nucleofected with empty vector (Figure 4). Activation of STAT1 by IFNγ was not affected by IE1 since similar levels of tyrosine 701‑phosphorylated STAT1 were observed after 30 minutes of IFNγ treatment in cells nucleofected with full length IE1, the Δ345–491 mutant (ΔAD), or empty vector (Figure 4). Similarly, no differences in accumulation of serine 727-phosphorylated STAT1 were observed (data not shown). We also found that translocation of STAT1 to the nucleus was not affected by IE1 when examined by immunofluorscence microscopy (Figure 5A). Consistent with the microscopy analysis, the accumulation of STAT1 protein in the nucleus of IE1 expressing cells was similar to that of cells expressing the Δ345–491 deletion mutant (ΔAD) and cells transfected with empty vector (Figure 5B). Thus, IE1 does not act by limiting the abundance of key IFNγ signal transduction molecules, nor does it disrupt STAT1 phosphorylation and nuclear translocation.

### 2.5. Type II Interferon-Induced Binding of STAT1 to GAS Elements Is Reduced in the Presence of IE1

**Figure 6:**
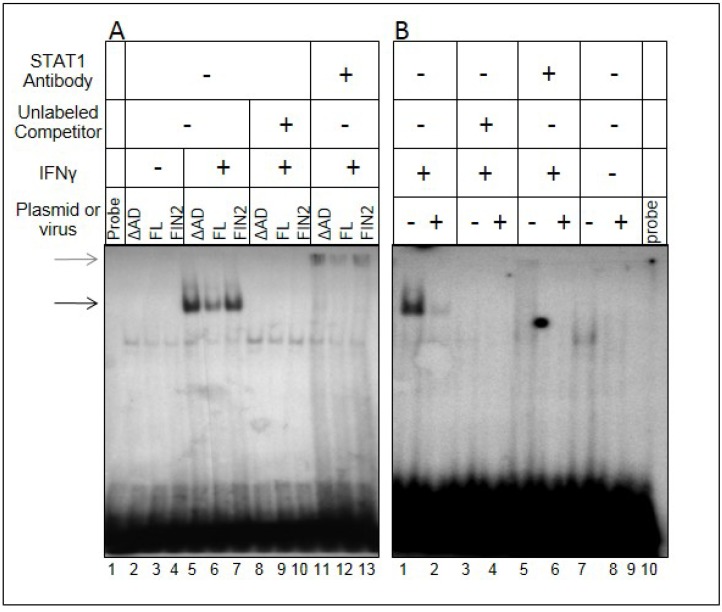
Reduced binding of STAT1 to GAS elements in the presence of IE1. Film image of an Electrophoretic Mobility Shift Assay shows a reduction in the amount of IRF1 GAS element probe that is shifted upon mixture with nuclear extracts from MRC5 cells ectopically expressing IE1 (**A**) and HCMV infected cells (**B**). In A, MRC5 cells were nucleofected with plasmid harboring full length IE1 (FL), the Δ345–491 C-terminal truncation IE1 (ΔAD) or empty vector (FIN2). At 24 hours after nucleofection, cells were exposed to 100 U/mL of IFNγ or left untreated. At 30 minutes after treatment, cells were solubilized and nuclear extracts were isolated as described in Materials and Methods. Nuclear extracts were mixed with the GAS element of the IRF1 gene with or without addition of unlabeled probe and with or without anti-STAT1 antibody. Resulting complexes were resolved by electrophoresis on a 4.5% native polyacrylamide gel. In a second experiment (**B**), MRC5 cells were exposed to 1 PFU per cell of the AD169 strain of HCMV or left uninfected. At 12 hours after infection, cells were solubilized and processed as described above. The black arrow indicates the shifted bands and the grey arrow indicates the supershifted bands.

We next determined whether STAT1 molecules in the nucleus were competent to bind the GAS element derived from the promoter of the IRF1 gene by EMSA. The full length IE1, the Δ345–491 mutant (ΔAD), and empty vector (FIN2) were nucleofected into MRC5 cells. These cells were either treated with IFNγ for 30 minutes or left untreated. Nuclear extracts from these cells were incubated with a radiolabeled 22mer IRF1 GAS element probe. The amount of probe shifted in nuclear extracts from IE1-expressing cells is diminished relative to extracts derived from empty vector- or Δ345–491-nucleofected cells (Figure 6A, compare lane 6 to lanes 5 and 7). These bands could be supershifted using a STAT1 antibody indicating that the shift was caused by a complex containing STAT1 (lanes 11–13). This demonstrates that there is reduced binding of STAT1 to GAS elements in the presence of IE1. Studies were also conducted with nuclear extracts from HCMV-infected cells at 12 hours after infection. We could detect only a minor STAT1-shifted probe band in HCMV infected cells compared to uninfected cells (Figure 6B, lane 2), which may be related to the much higher levels of IE1 in infected cells relative to cells nucleofected with full length IE1 (data not shown). Together, these data indicate that expression of HCMV IE1 is sufficient to impair STAT1 binding to GAS elements, and that this activity requires the C-terminal region of IE1 including residues 344 to 491. These data also suggest that the impairment of functional STAT1 dimer binding to GAS elements at 12 hours after infection is a result of IE1 expression. 

IE1 has been reported to antagonize HDAC activity [36]. Although HDAC activity is typically associated with transcriptional repression, it is associated with transcriptional activation of ISGs [48]. It is tempting to speculate that antagonism of HDAC activity by IE1 is linked to disruption of interferon signaling. Further implicating a role for chromatin organization is that the C-terminal region of IE1 includes the region previously shown to be required for chromatin tethering [38]. However, this possibility is difficult to reconcile with the observation that IE1 disrupts STAT1-binding to a GAS element probe used in our EMSA assays, which is not dependent on chromatin structure. We therefore propose that a novel function of IE1 impairs STAT1 binding to target GAS elements.

### 2.6. STAT1 Fails to Co-Immunoprecipitate with IE1

**Figure 7:**
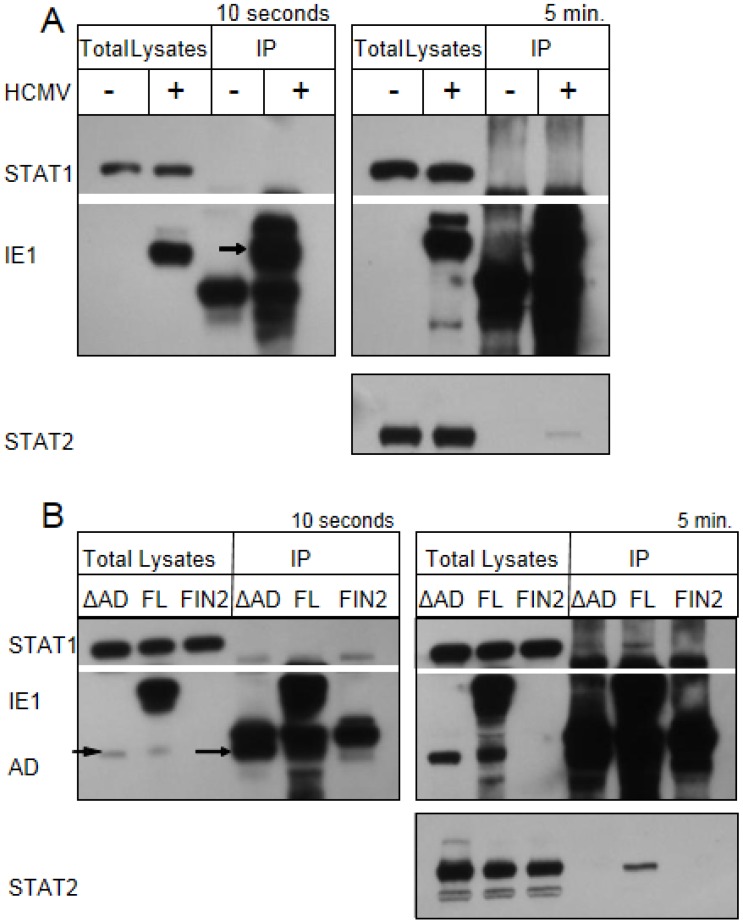
IE1 does not interact with STAT1 by co-immunoprecipitation. Film images of electrophoretically separated cell lysates and proteins isolated by immunoprecipation. (**A**) MRC5 cells were exposed to HCMV AD169 at 3 PFU per cell or left untreated. At 12 hours after infection cells were treated with 100 U/mL of IFNγ for 30 minutes. The cells were harvested and solubilized. 350 µg of total protein from each sample was reacted with anti‑IE1 antibody (mouse monoclonal Mab810). The isolated proteins along with the 10 µg total lysates were resolved by denaturing PAGE and transferred to a nitrocellulose membrane. The membrane was reacted with antibodies for STAT1, IE1 and STAT2 and the antibody reactive bands were visualized by chemiluminescence. The left panel shows films exposed for 10 seconds and the right panel shows films exposed for 5 minutes. The arrow indicates the position of the isolated, full length IE1. (**B**) MRC5 cells were nucleofected with full length IE1 (FL), the Δ345–491 C-terminal truncation IE1 (ΔAD) or empty vector (FIN2). 48 hours after nucleofection cells were exposed to 100 U/mL of IFNγ for 30 minutes. The preparation of cell lysates, immunoprecipitations, PAGE and western hybridization and detection were carried out as above. The left panel shows films exposed for 10 seconds and the right panel shows films exposed for 5 minutes. The short and long arrows indicate the position of the Δ345–491 IE1 in the total lysate and immunoprecipitates respectively.

In the EMSA studies, the size of the shifted band in the presence or absence of STAT1 antibody is the same whether or not IE1 is present. This argues against a scenario in which IE1 acts by directly binding to STAT1-bound GAS elements. In order to test this directly, we carried out co‑immunoprecipitation studies. MRC-5 fibroblast cells were exposed to 3 PFU per cell of the AD169 strain of HCMV. At 12 hours after infection, cells were treated with interferon for 30 minutes. Cells were solubilized and IE1 was isolated using an antibody that recognizes an epitope in exon 2. Under these conditions, we found no evidence of an interaction between STAT1 and IE1 (Figure 7A). Similarly, STAT1 failed to specifically co-immunoprecipitate with either the full length or the Δ345–491 mutant of IE1 in nucleofection studies (Figure 7B, left panel). We did observe a small amount of STAT1 isolated from cells expressing both the full length IE1 and the C-terminal mutant upon longer exposure (right panel). However, in several replicate experiments, similar amounts of STAT1 were also observed in immunoprecipitations performed using lysates of empty-vector-transfected cells (data not shown). We therefore conclude that the small amount of STAT1 isolated upon isolation of IE1 and the Δ345–491 mutant IE1 represents nonspecific binding. These studies suggest that IE1does not require a sustained interaction with STAT1 to disrupt IFNγ signaling. 

**Figure 8:**
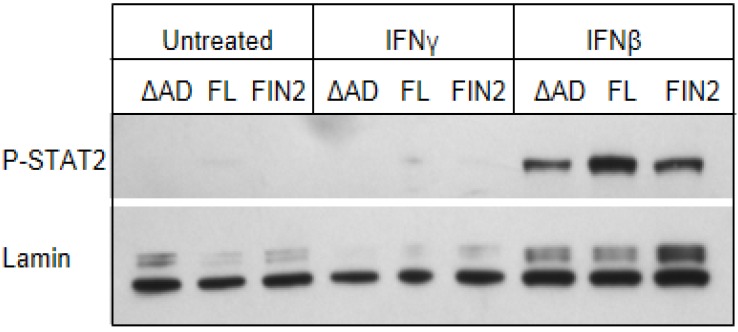
Phosphorylated STAT2 does not accumulate in the nucleus of IFNγ-treated MRC-5 cells. Film images of electrophoretically separated nuclear lysates reacted with antibodies to p-STAT2 and Lamin (loading control). MRC5 cells were nucleofected with full length IE1 (FL), the Δ345–491 C-terminal truncation IE1 (ΔAD) or empty vector (FIN2). At 72 hours after nucleofection, cells were exposed to IFNγ or IFNβ for 30 minutes or left untreated. Cells were harvested and nuclear and cytoplasmic fractions were separated. Equal amounts of protein from the nuclear lysates were subjected to electrophoresis in a denaturing polyacrylamide gel. Proteins were transferred to nitrocellulose sheets and reacted with the indicated antibodies.

A blockade in IFNγ-mediated regulation of several genes is observed in murine cytomegalovirus (MCMV) infected macrophages [25], and the MCMV M27 protein has been shown to disrupt IFNγ signaling through a novel, STAT2-dependent mechanism [26]. Therefore, we examined whether STAT2 co-immunoprecipitates with IE1 in IFNγ treated human fibroblasts. As expected based previously published studies [21,22,23,24], we did observe an interaction of STAT2 with the full length IE1 in total lysates of nucleofected cells and HCMV infected cells. Similar to previously published results, this interaction maps to the C-terminal region of IE1 and the Δ345–491 mutant does not appear to interact with STAT2 under these conditions (Figure 7B). In addition, very little or no phosphorylated STAT2 accumulated in nuclei of MRC5 human fibroblasts in response to IFNγ treatment, though phosphorylated STAT2 was observed in nuclei after IFNβ treatment (Figure 8). Our findings raise the possibility that IFNγ signaling is different in murine and human fibroblasts. Specifically, it seems that murine, but not human fibroblasts, utilize phosphorylated STAT2 to transduce and amplify signals by IFNγ. Together, these studies suggest that IE functions via a novel mechanism in the nucleus to interfere with the type II IFN signal transduction pathway.

An important question raised by our studies is whether IE1 employs the same strategy, or entirely different strategies to disrupt type I and type II interferon signaling. Relevant are the following observations: (i) co-immunoprecipation studies using total lysates indicate an interaction between the HCMV IE1 acidic domain and STAT2 in human cells consistent with other studies ([21,22,23,24] and this report), and IE1 and STAT2 colocalize in ND10 structures and metaphase chromosomes [23]; (ii) in human fibroblasts, phophorylated STAT2 does not accumulate in the nucleus upon IFNγ treatment ([24] and this report); (iii) there are conflicting reports of an interaction between IE1 of the Towne strain and STAT1 [21,23] and we were unable to find compelling evidence for a specific interaction between IE1 of strain AD169 and STAT1; and (iv) nevertheless, IE1 expression interferes with binding of STAT1 molecules to GAS elements. Based on these observations, it is tempting to conclude that an interaction between IE1 and STAT2 is necessary and sufficient to interfere with type I IFN signaling. However, such an interaction does not readily account for the activity of IE1 in attenuating binding of STAT1 molecules to GAS elements in response to type II interferon. On the other hand, the ability of IE1 to disrupt STAT1 binding to target DNA elements through an indirect mechanism could impede signals transduced by both type I and type II interferons. We suggest further studies are needed to resolve the role of IE1 in these signaling pathways.

Many DNA viruses encode proteins that target the JAK- STAT signaling pathway including adenovirus E1A [49,50], human papilloma virus (HPV) E7 protein [51], hepatitis B virus (HBV) terminal protein [52], and polyoma virus large T antigen [53]. Among the herpesviruses, the EBV immediate early protein BZLF1 has been shown to interfere with STAT1 phosphorylation and nuclear translocation in response to IFNγ, as well as IFNγ receptor expression [54]. Similarly, herpes simplex virus (HSV) was shown to interfere with type I IFN signaling by decreasing the levels of several signaling molecules, including JAK1 and STAT2, which was in part mediated by the virion host shutoff protein encoded by *UL41* [55,56], by inducing suppressor of cytokine signaling-3 (SOCS3) [57], and by the activity of the ICP27 protein in inhibiting STAT1 phosphorylation and nuclear translocation [58]. HSV also targets the IFNγ pathway by modifying the IFNγR1 through the activity of the US3 and UL13 protein kinases, and indirectly by the activities of virion host shutoff protein [59]. With the partial exception of HSV virion host shutoff protein, all of these viruses target early events in the signaling pathway, especially STAT1 phosphorylation and nuclear translocation, to attenuate IFN mediated signaling. Our findings indicate that HCMV does not target these proximal events in IFN signaling but rather it reduces STAT1 binding to target promoter elements in the nucleus. Elucidating the mechanism and consequences of this novel immune evasion strategy may provide important insights into the pathogenesis of HCMV infections and the regulation of interferon-induced gene expression.

## 3. Experimental Section

### 3.1. Cells and Viruses

Human embryonic lung fibroblasts (MRC5) were obtained from American Type Culture Collection (ATCC, Manassas, VA, USA) and maintained in ATCC-recommended complete media. Human fibrosarcoma cell line 2C4 was a kind gift from George Stark (Cleveland Clinic Lerner Research Institute, Cleveland, OH, USA) [34]. 2C4 cells were maintained in DMEM supplemented with 10% FBS, Glutamine and Sodium Pyruvate. HCMV strain AD169 was obtained from ATCC and propagated in MRC5 cells. Viral titers were determined by standard plaque assay [60].

Plasmids and vectors: Construction of the AD169 cDNA library is described elsewhere [33]. cDNA clones containing different truncated versions of the IE1 gene were selected from the cDNA library. The following clones were used in this study and harbor viral gene sequences with the indicated genomic coordinates: pie639 (173157-171814), pie535 (172658-171814), pie836 (172436-171814). The sequences specifying the FLAG epitope tag were inserted at the 5' end of each truncation mutant by replacing the Nde I–Eco R1 fragment of each clone with the Nde I–Eco R1 fragment from the pCMV3TAG vector (Stratagene, La Jolla, CA, USA) that includes both the CMVIE1 promoter sequence and the sequences specifying the FLAG tag. The resultant plasmids specified 5' FLAG tag sequences in frame with different truncated versions of IE1. For the C-terminal deletion mutant, a Bgl II fragment from cDNA clone pie622 (a full length IE1 cDNA clone), was inserted into the pCMV3TAG vector containing a FLAG tag at the 5' end in the same coding frame. The genomic coordinates for the C-terminal deletion mutant are (173705-173626 + 173511-173327 + 173156-172377). To generate a full length *UL123* gene sequence in frame with *FLAG* sequences, the IE1 cDNA was PCR amplified from pie622 using the following primers: 'AGAGTGACTCACCAAGCTTGACACGATG and CGAGGCTGATCAGCTTAATTAACC. The PCR products were digested with Hind III and Pac I and inserted into HindIII- and PacI-digested pCMV3TAG vector. To generate a UL123 construct missing exon 2 in frame with *FLAG* sequences, pie622 was used as template to PCR amplify the appropriate gene regions using the following primers: CCTTCCTCCAAGCTTCCACGGCC and CGAGGCTGATCAGCTTAATTAACC which begins at genomic coordinate 173,645. The PCR products were digested with Hind III and Pac I and ligated into HindIII and PacI digested pCMV3TAG vector.

### 3.2. Transfection, Nucleofection and Infection

40,000 2C4 cells were plated per well of a 12-well plate. Within 24 hours of plating cells were transfected with the cDNA clones specifically using the Fugene HD transfection reagent (Roche, Indianapolis, IN, USA) according to manufacturer’s recommendations (1 µg plasmid DNA/well with 3 µL of transfection reagent) [61]. Typically, this procedure resulted in 40%–50% transfection efficiency (data not shown). Transfections were optimized to reduce non-specific cellular interferon responses [61]. For nucleofections, 1.5 × 10^6^ MRC5 cells were resuspended in 100 µL of basic nucleofector solution for primary fibroblasts with 3 µg of plasmid DNA and nucleofected using U23 program in the nucleofector device (Amaxa, Cologne, Germany). After nucleofection cells were immediately distributed equally in three wells of a six-well cluster. Nucleofection and transfection efficiencies were determined by immunofluorescence microscopy analysis of IE1 expression and/or immunofluorescence microscopy analysis of GFP expression in parallel transfections using plasmid pEGFP-N3 (Clontech, Mountainview, CA, USA). Nucleofection resulted in greater than 90% transfection efficiency (data not shown). For infections, confluent MRC5 cells in 75 cm^2^ tissue culture flasks were exposed to one or three plaque forming unites (PFU) per cell of HCMV strain AD169 and harvested 24 hours post infection. Wherever indicated, cells were treated with 100 U/mL of human recombinant IFNβ or IFNγ (Chemicon (now EMD Millipore), Billerica, MA, USA). All interferon treatments were performed 24 hours after transfection or nucleofection. 

### 3.3. Immunoblotting and Immunoprecipitation

Infected or nucleofected cells were rinsed with versene (PBS + 1 mM EDTA) to dislodge cells. No trypsin was used in these studies. Harvested cells were pelleted by centrifugation at 1500 rpm for 5 minutes and resuspended in lysis buffer (25 mM Tris, 150 mM NaCl, 1% NP40, 10 mM NaF, 0.1 mM NaVO_4_, 1 mM EDTA and 1% (vol/vol) protease inhibitor cocktail (Sigma, St. Louis, MO, USA). The cells were then sonicated in a Misonix (Farmingdale, NY, USA) Cuphorn Sonicator for 100 seconds (5 s on, 5 s off) at setting 5. Sonicated cells were centrifuged at 13,000 rpm for 5 minutes to pellet insoluble material. Protein was quantified using Bradford dye (Bio-Rad, Hercules, CA, USA) according to manufacturer recommendations. For immunoprecipitation, cell lysates were precleared by incubating with protein A agarose beads for 30 minutes at 4 °C. The precleared lysates were incubated with primary antibody at 4 °C overnight followed by incubation with Protein A beads for 2 hours at 4 °C. The isolated complexes were rinsed four times in lysis buffer and eluted by boiling in 2× Laemmli buffer with 5% β-mercaptoethanol. Proteins were separated by SDS‑PAGE, transferred to a nitocellulose membrane (Whatman (now GE Healthcare Bio-Sciences), Pittshburgh, PA, USA), reacted with primary antibodies overnight, exposed to HRP- conjugated secondary antibody, and detected by ECL (Amersham (now GE Healthcare Bio-Sciences), Pittshburgh, PA, USA). Antibodies to the following were used: JAK1 (Upstate (now EMD Millipore), Billerica, MA), JAK2 (Santacruz Biotechnology, Dallas, TX, USA), STAT1 (Santacruz), STAT2 (Cell Signaling, Danvers, MA, USA), phosphotyrosine STAT 1 (tyr 701, Cell Signaling), phosphoserine STAT1 (ser 727, Cell Signaling), FLAG (Sigma), Glyceraldehyde 3-phosphate dehydrogenase (GAPDH) (Chemicon), phosphoSTAT2 (Upstate), IE1 exon 2 antibody (Chemicon), IE1 exon 4 antibody p63-27 (gift of Dr. William Britt, University of Alabama at Birmingham, Birmingham, AL, USA).

### 3.4. Flow Cytometry

2C4 cells were rinsed with versene and nonezymatically dislodged from wells at 24 hours after IFN treatment. Cells were resuspended in flow buffer (PBS with 1% FBS) and reacted with FITC-conjugated anti-CD2 antibody (Dako, Carpinteria, CA, USA) according to manufacturers recommendations. Cells were rinsed with flow buffer and CD2 fluorescence was measured in a Facscalibur Flow Cytometer (Becton Dickenson, Franklin Lakes, NJ, USA). The data were analyzed with the aid of CELLQuest 3.0 software (Becton Dickinson) [62].

### 3.5. Real Time RT-PCR

Total RNA from MRC-5 cells was isolated using Trizol (Invitrogen (now Life Technologies), Grand Island, NY, USA) according to the manufacturer’s protocol. Next, 1 µg of total RNA from each sample was used to perform first strand cDNA synthesis using Superscript reverse transcriptase (RT) (Invitrogen) as per manufacturer’s recommendations. The cDNA was diluted 1:4 and 8 µL was used per real-time polymerase chain reaction (PCR) of 25 µL total volume. For reactions using SYBR green, the primers used for CIITA and GAPDH (endogenous control) are described elsewhere [63]. For reactions using Taqman^TM^ the following primer and probe sets were used: CIITA-Hs00172106_mL, IRF1-Hs00233698_mL and 18SrRNA-4310893E (Applied Biosystems (now Life Technologies), Grand Island, NY, USA). All Real-Time reactions were set up in triplicate in 96 well format and analyzed in an ABI prism 7900HT Real-Time instrument. Fold change in CIITA or IRF1 expression in IFN treated samples compared to untreated samples was calculated using the 2^−ΔΔCt^ method after normalizing to the endogenous control (either GAPDH or 18SrRNA) [64].

### 3.6. Microscopy

Nucleofected or transfected cells were plated on 12 mm coverslips in 24 well plates. Wherever indicated cells were exposed to 100 U/mL IFN for 30 minutes. Cells were rinsed with PBS and fixed in methanol for 1 hour on ice. The cells were then air dried and stored at 4 °C. Cells were rehydrated in PBS for 5 minutes and incubated in blocking buffer (PBS, 10% human serum, 1% BSA) for 1hr. This was followed by incubation with primary antibody in blocking buffer overnight at 4 °C. Cells were rinsed three times for 20 minutes with PBS. This was followed by exposure to fluorescently labeled secondary antibody for 1 hour at 4 °C. Excess secondary antibody was rinsed off with three rinses with PBS. The coverslips were allowed to air dry and then were mounted on glass slides using the Prolong antifade kit (Molecular Probes (now Life Technologies), Grand Island, NY, USA). Microscopy was performed using a Zeiss LSM 510 instrument. Each fluorescent dye was scanned separately to avoid any spectral overlap. Images were prepared using Zeiss LSM 5 Software, Version 3.5 [65]. The following primary antibodies were used: anti-IE1 exon 2 antibody (Chemicon), anti-IE1 exon 4 antibody p63-27 (gift of Dr. William Britt), anti-PML (Santacruz), anti-STAT1 (Santa Cruz).

### 3.7. Electrophoretic Mobility Shift Assay (EMSA)

MRC5 cells were plated at a density of 1 × 10^6^ cells per 75 cm^2^ flask post nucleofection. Cells were exposed to 100 U/mL IFNγ for 30 minutes or left untreated. Cells were rinsed with PBS, dislodged from the plates, and nuclear extracts were prepared using Nuclear extract kit (Activ Motif, Carlsbad, CA, USA). A 22mer Interferon Response Factor 1 (IRF1) GAS element probe (GATCGATTTCCCCGAAATCATG ) was radiolabeled with γ [P]^32^ ATP using T4 polynucleotide kinase enzyme (Invitrogen) according to the manufacturer’s recommendations. 4 µg of nuclear extract was incubated with 1× binding buffer, 0.5 µg poly dI:C, 0.1 µg poly L-lysine and 2ng of labeled probe for 15 minutes at room temperature. For competition assays, a 200-fold excess of unlabeled probe was added to the reaction mix. For supershift studies, 5 µg of rabbit polyclonal anti STAT1 antibody (Santacruz) was added to the reaction and incubated for 15 minutes at room temperature prior to adding the labeled probe. The reactions were resolved on a 4.5% native polyacrylamide gel in 0.5× TBE buffer and visualized by autoradiography.

## 4. Conclusions

The major findings of this report are as follows: (i) IE1, the immediate early transcriptional transactivator of HCMV, was identified as a candidate protein that disrupts IFN signaling by screening a HCMV cDNA library using the 2C4 reporter cell line and primary human fibroblasts; (ii) Using a series of truncation mutants we assigned this function of IE1 to the C-terminal 147 residues of the IE1 protein; (iii) IE1-mediated disruption of IFNγ signaling appears to be independent of IE1-mediated ND10 disruption; (iv) IE1 does not inhibit the proximal events of type II IFN signaling; and (v) Although IE1 does not appear to directly interact with STAT1, expression of IE1 interferes with STAT1 binding to target DNA elements. Together our data indicate that a novel mechanism is employed by IE1 to interfere with the IFNγ signal transduction pathway. In earlier studies, Miller *et al.* [28] have reported a reduction in STAT1 homodimer binding to GAS elements starting at 12 hours of infection and continuing up to 72 hours of infection. Furthermore, LeRoy *et al.* reported a defect in CIITA induction by IFNγ that occurs downstream of STAT1 nuclear translocation in infected cells [27]. Our results suggest that these phenomena are mediated by IE1. Although only cells of the immune system including NK cells and T cells produce IFNγ, most or all nucleated cells in the body can express IFNγ receptors and thus elaborate antiviral responses when stimulated by IFNγ. It is known that during a primary infection CD8^+^ T cells play an important role in controlling the spread of the virus. During this time it may be advantageous for the virus to be able to dampen signaling induced by IFNγ, which is produced abundantly by CD8^+^ T cells. This could not only counteract IFNγ–mediated antiviral responses, but also interfere with IFNγ-induced expression of MHC class I and MHC class II molecules, assembly of the immunoproteasome [66] and the presentation of viral antigens. Therefore, viral disruption of IFNγ-mediated signaling may promote virus replication by attenuating both intrinsic cellular defense mechanisms and adaptive immune responses.
